# Determinants of podoconiosis at the age of 15 years and above at Dera Woreda, South Gondar zone, Northwest Ethiopia

**DOI:** 10.1186/s12889-025-24723-8

**Published:** 2025-10-06

**Authors:** Animut Takele Telayneh, Alebachew Mulate Tadesse, Habtamu Temesgen, Samuel Derbie Habtegiorgis, Moges Wubie Aycheh

**Affiliations:** 1 Department of Public Health, College of Medicine and Health Sciences, Debre Markos University, Debre Markos, Ethiopia; 2 Dera Woreda Health Office, Anbesamie, South Gondar Zone Ethiopia; 3 Department of Human Nutrition, College of Medicine and Health Sciences, Debre Markos University, Debre Markos, Ethiopia

**Keywords:** Podoconiosis, Determinants, Neglected tropical disease, Dera woreda, Ethiopia

## Abstract

**Background:**

Podoconiosis is a chronic non-infectious neglected tropical disease that predominantly affects barefoot individuals who are exposed to red clay soil. Podoconiosis is characterized by bilateral swelling of the lower legs, which has a wide range of impacts, including chronic morbidity, disability, and social stigma. It is the most common neglected public health problem across productive age groups, but not among children under the age of 15. The purpose of this study was to identify the determinants of podoconiosis among people 15 years and above at Dera Woreda, South Gondar Zone.

**Methods:**

A community-based unmatched case-control study design was employed using 504 study participants. The data was collected using a pre-tested structured interview administered questionnaire. EpiData version 3.1 was used for data entry, which was then exported to SPSS version 25 for cleaning, recoding, and further analysis. Variables with a p-value < 0.20 in binary logistic regression were selected for multivariable logistic regression analysis. To identify determinants of podoconiosis, variables with a p-value < 0.05 and 95% CI corresponding AOR were used.

**Results:**

In this study, 504 participants (169 cases and 335 controls) were included with a response rate of 98.2%. The study participants’ median age was 45 years (IQR = 35.25, 56.0). Older age [AOR: 3.05 (95% CI: 1.66, 5.60)], males [AOR: 2.03 (95%CI: 1.16, 3.55)], ≤ 1000birr monthly income [AOR: 9.16 (95% CI: 4.97, 16.87)], haven’t owned pair of shoes [AOR: 3.28 (95% CI: 1.77, 6.08)], didn’t wash feet daily [AOR: 2.93 (95% CI: 1.22, 7.02)], and family history [AOR: 21.67 (95% CI: 9.21, 51.01)] were identified determinants of podoconiosis.

**Conclusions:**

This study identified podoconiosis determinants as older age, male gender, low monthly income, not owning a pair of shoes, not washing their feet daily, and family history. Improving foot hygiene practices, owning shoes, and implementing community-led surveillance and screening activities are all necessary to prevent podoconiosis in the study setting. Improving and strengthening behavioral change through health education and promotion is required to prevent this neglected tropical disease across the nation.

**Supplementary Information:**

The online version contains supplementary material available at 10.1186/s12889-025-24723-8.

## Introduction

Podoconiosis is a chronic, non-infectious neglected tropical disease that predominantly affects agrarian people who work barefoot in red volcanic soil [1–4]. It is characterized by bilateral swelling of the lower legs with mossy and nodular skin changes, which causes significant disability [3, 5–7]. The disease resulting from a complex interaction between genes and the environment has not been well addressed; it continues to develop over many years [4, 5, 8–10]. The World Health Organization (WHO) implemented the Neglected Tropical Diseases (NTDs) program to prevent, control, eliminate, or eradicate tropical diseases including podoconiosis by 2030 [11–13]. Globally, the disease occurs in highlands in tropical Africa, Central America, and northern India [2, 3, 14]. The podoconiosis disease worldwide affects about 4 million people [8, 14, 15]. The prevalence of podoconiosis in Sub-Saharan Africa was 2.66%, with geographic variations of 0.07% in Rwanda [2], 8.08% in Cameroon [16], 4.52% in Uganda [17], and 3.87% in Kenya [18].

In Ethiopia, podoconiosis is estimated to affect more than 1.5 million cases, with 35 million people at risk in 345 districts (Woredas). The national prevalence was 4%, with 8.3% in the Southern Nation and Nationality People, followed by 4% in Oromia and 3.9% in Amhara regions, which had the highest prevalence in 2015 [19]. Podoconiosis has a wide range of impacts, including substantial economic and social burdens such as dependency due to loss of productivity, high stigma at school and workplace, and exclusion from social linkage like marriage [14, 20, 21]. It is a modern and silent public health problem, with hundreds of thousands of people suffering from this skin disease [20]. Gender, illiteracy, low monthly income, marital status, age, family size, mossy lesions, and open wounds are all common risk factors for podoconiosis [1, 6, 22–25]. Similarly, family history of leg swelling, distance to the health facility, walking barefoot, climate conditions, and soil type have all been associated with podoconiosis [26–30].

Podoconiosis NTDs intervention plan includes prevention through consistent use of footwear (shoes and socks) starting at an early age, regular foot hygiene, covering housing floors, lymphedema management, and treatment of acute attacks to reduce further exposure to the irritant soil [1, 9, 14, 31–33]. In 2013, Ethiopia established its first National Master Plan for the control, elimination, and eradication of nine priority NTDs, including podoconiosis [34]. However, the prevention and treatment of podoconiosis are not given sufficient attention by different stakeholders as a disease of public health importance, resulting in a lack of awareness, little attention by the government, unavailability of prevention and treatment service centers, and lack of updated information on the prevalence of podoconiosis in Ethiopia [14, 28, 35].

Despite previous decades of efforts to prevent NTDs in Ethiopia [35, 36], the most common barriers to the provision of podoconiosis care, treatment, and prevention measures remain an inadequate surveillance system, a dysfunctional community referral, and poor awareness [15, 35, 37]. Determinants of podoconiosis have been addressed in Ethiopia’s previous studies [20, 24, 26, 28, 38–40], but there is still scarce scientific evidence to address this devastating neglected public health problem. Evidence revealed that the incidence of podoconiosis increased in people who lived in places with irritating red clay soils, high altitudes > 1500 m above sea level, yearly rainfall > 1000 millimeters, and temperatures > 20 °C [41, 42]. This study was conducted in Dera Woreda, which has a high number of podoconiosis cases and has received podoconiosis prevention and treatment services. Moreover, altitude of 1500–2600 m, annual rainfall of 1000–1500 millimeters, soil types (25% red clay), and agroecological conditions with a majority of agrarian communities are enabling factors of podoconiosis endemic in the Woreda [43]. In addition to the evidence gaps, these are driving factors to urge us to conduct a study in this setting. This finding is helpful for healthcare workers, decision-makers, and other stakeholders to plan and implement innovative intervention strategies against this neglected tropical disease prevention and control measures. Hence, this study aimed to identify the determinant factors of podoconiosis in Dera Woreda South Gondar Zone, Northwest Ethiopia.

## Methods

### Study design, setting, and population

A community-based, unmatched case-control study was conducted. From April 1–30, 2023, the study was conducted at Dera Woreda South Gondar Zone, Amhara region. It is located 42 km from Bahir Dar Amhara Regional State and 608 km from Addis Ababa, Ethiopia’s capital. The Woreda has a total population of 310,038 people, of which around 177,856 (57.4%) are 15 years and above. Dera Woreda soil type is 25% red clay, 65% sand, and 10% clay, with an annual rainfall of 100–1500 millimeters at an altitude ranging from 1500 to 2600 m above sea level. Dera Woreda has 39 kebeles (the lowest governmental administrative unit) with 11 health centers and 36 health posts that provide primary healthcare services to the community [43].

### Eligibility criteria of study participants

People who were 15 years and above lived in the study area for more than 10 years in selected kebeles during the study period were included in the study. The specific criteria were:

- Cases: An individual who has been diagnosed and registered as podoconiosis disease by a trained nurse or a health extension worker (HEW) using the criteria below (Table 1) [44].
- Controls: An individual with no past or current history, signs, and symptoms or has not been diagnosed podoconiosis by a trained nurse or HEW.

**Table 1:** Clinical criteria used for diagnosis of podoconiosis cases

Major criteria	Minor criteria
1. Below-knee lymphedema	1. Nodules
2. Residing in an endemic area during the development of lymphedema	2. Toe fusion
3. Barefooted waking for prolonged periods of time (more than 10 years)	3. Both feet affected
4. Mossy feet in a slipper pattern	4. Positive Stemmer’s sign (i.e., the thickened fold of skin at the base of the second toe that can be pinched and lifted. This test is positive when the skin cannot be lifted, showing the presence of lymphedema)
	5. Burning sensation of the feet
	6. Family history of podoconiosis
	7. The problem started in the first three decades of life
Definition podoconiosis 1. Definitive podoconiosis: 3 majors; or 2 majors plus 2 minors; or 1 major plus 5 minor criteria 2. Probable podoconiosis: 2 majors; or 1 major plus 2 minors; or 5 minor criteria	

### Sample size determination and sampling procedure

**Fig. 1:**
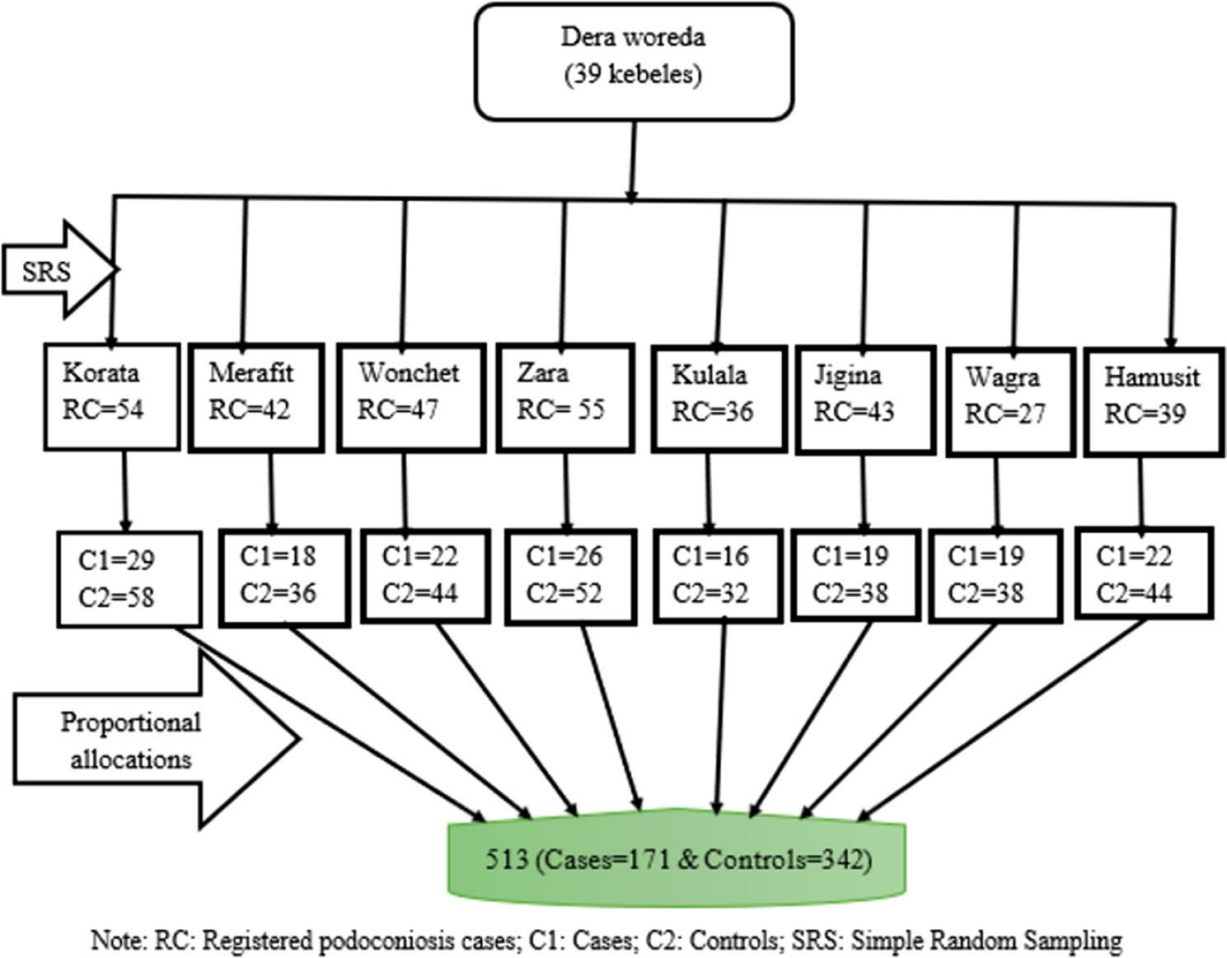
Sampling procedure of determinant of podoconiosis among the age of 15 years and above in Dera Woreda, Northwest Ethiopia, 2023

The sample size was calculated using Epi-Info version 3.1 software, using a previous study conducted in the Dano district, Central Ethiopia [20]. This is estimated using the following assumptions: a 6.4% proportion of controls washing with water only, an adjusted odds ratio (AOR) of 3.68, a confidence interval (CI) of 95%, a power of 80%, and a case/control ratio of 1:2. After taking into account two design effects and a 10% non-response rate, the final sample size yielded 513 (171 cases and 342 controls). In Dera Woreda, there are 39 kebeles with diagnosed podoconiosis cases; about 8 kebeles (20%) were selected by a simple random sampling technique. In these selected kebeles, 343 podoconiosis cases were registered in the International Orthodox Christian Charities registration book and received their treatment and care. Cases for each kebele were allocated proportionally based on size, while controls were drawn from the community to the nearest neighbor of the selected case. They share common socio-demographic, cultural, and environmental exposures to podoconiosis (i.e. water source, soil type, housing condition, shoe-wearing habit, etc.). The cases were selected using a computer-generated simple random sampling technique. Similarly, controls were selected using a simple random lottery method among those who resided in the nearest house to the cases. If there were two or more people with ≥ 15 years of control in one house, we used the lottery method to participate in the study (Fig. 1).

### Operational definition

- Sufficient water availability: This is defined as having at least 20 L of water per person per day within a round trip walking distance of 30 min or 1 km distance from the user’s dwelling for drinking and maintaining personal hygiene [4, 45].
- Washing feet daily: This is defined as washing their feet one or more times daily with or without soup.

### Data collection tool, procedure, and quality assurance

The tool was prepared in English, then translated into the local language “Amharic” and back to English to maintain consistency. A structured face-to-face interview-administered questionnaire was used to collect the data, which included socio-demographic characteristics, behavioral, exposure, and practice-related factors (Supporting Table 1). Eight diploma nurse data collectors and two MSc nursing supervisors participated. After enrolling and interviewing the case, the control was recruited from the nearest unaffected households. The quality of the tool was assured by using 5% of the questionnaires that were pre-tested out of the selected kebeles, one-day training for data collectors and supervisors was given by healthcare professionals with experience in podoconiosis diagnosis, treatment, and care, and close monitoring and supervision was held during the entire data collection period. Incomplete or missed data is rectified on a daily basis, and errors are controlled using a double-entry process.

### Data processing and analysis

Data were entered using EpiData version 3.1 and then exported into SPSS version 25 for cleaning, recoding, and further analysis. The descriptive findings were presented by using frequency tables, median, and ranges. In binary logistic regression analysis, variables with a p-value ≤ 0.20 were selected for multivariable logistic regression analysis. Multicollinearity was checked between variables. The Hosmer-Lemeshow goodness-of-fit test (*p* = 0.704) was used to assess model fitness. In a multivariable model, variables with a p-value < 0.05, 95% CI, and AOR were used to identify statistically significant determinants of podoconiosis.

## Results

### Socio-demographic characteristics

In this study, 504 participants (169 cases and 335 controls) were included with a response rate of 98.2%. The study participants median age was 45 years (IQR = 35.25, 56.0). About 60 (35.5%) cases and 216 (64.5%) controls were between the ages of 15 and 45 years. One hundred seventeen (69.2%) cases and 190 (56.7%) controls were male participants. In all cases, 169 (100%) and 332 (99.4%) controls were Orthodox religious followers, while 98 (58.0%) cases and 266 (79.4%) controls were married (Table 2).

**Table 2:** Socio-demographic characteristics of podoconiosis at the age of 15 years and above in dera woreda, Northwest ethiopia, 2023 (*n* = 504)

Variables		Cases *n* (%)	Controls *n* (%)	Total *n* (%)
Age	15–45 years	60 (35.5)	216 (64.5)	276 (54.8)
	> 45 years	109 (64.5)	119 (35.5)	228 (45.2)
Sex	Male	117 (69.2)	190 (56.7)	307 (60.9)
	Female	52 (30.8)	145 (43.3)	197 (39.1)
Marital status	Single	22 (13.0)	30 (9.0)	52 (10.3)
	Married	98 (58.0)	266 (79.4)	364 (72.2)
	Divorced	25 (14.8)	26 (7.8)	51 (10.2)
	Widowed	24 (14.2)	13 (3.8)	37 (7.3)
Education	No formal education	134 (79.3)	220 (65.7)	354 (70.2)
	Formal education	35 (20.7)	115 (34.3)	150 (29.8)
Occupation status	Farmer	141 (83.4)	297 (88.6)	438 (86.9)
	Merchant	22 (13.0)	25 (7.5)	47 (9.3)
	Daily labor	6 (3.6)	13 (3.9)	19 (3.8)
Monthly income	≤ 1000 birrs (≤ 7.16 USD)	147 (86.9)	127 (37.9)	274 (54.4)
	> 1000 birrs (> 7.16USD)	22 (13.1)	208 (62.1)	230 (45.6)

### Behavioral, practice, and exposure-related characteristics

In this study, 85 (50.3%) cases and 183 (54.6%) controls believe podoconiosis disease is preventable by using appropriate preventive measures. Only 65 (38.5%) cases and 243 (72.5%) controls have their own pairs of shoes, while the majority of 50 (76.9%) cases and 191 (78.6%) controls were wearing shoes during the interview. About 59 (89.8%) cases and 116 (47.7%) controls in the study wore shoes that were older than 15 years. Only 40 (26.7%) cases and 138 (41.2%) controls have sufficient water. A minority of the respondents, 21 (12.4%) of cases and 120 (35.5%) of controls were washed their feet daily (Table 3).

**Table 3:** Behavioral, practice, and exposure-related characteristics of podoconiosis at the age of 15 years and above in dera woreda, Northwest ethiopia, 2023 (*n* = 504)

Variables		Cases *n* (%)	Controls *n* (%)	Total *n* (%)
Podoconiosis preventable	No	84 (49.7)	152 (45.4)	236 (46.8)
	Yes	85 (50.3)	183 (54.6)	268 (53.2)
Ways of podoconiosis prevention (268)	Wearing shoe	44 (51.8)	83 (45.4)	129 (47.9)
	Washing feet regularly	29 (34.1)	51 (27.9)	81 (30.1)
	Avoiding married affected people	2 (2.4)	28 (15.3)	29 (10.8)
	Avoiding contact with affected people	10 (11.7)	21 (11.4)	30 (11.2)
Why not prevented (236)	Genetics	34 (40.5)	75 (49.3)	111 (47.2)
	No drugs to cure	32 (38.1)	45 (29.6)	77 (32.8)
	Not seen cured people	18 (21.4)	32 (20.1)	47 (20.0)
Have owned a pair of shoes	No	104 (61.5)	92 (27.5)	196 (38.9)
	Yes	65 (38.5)	243 (72.5)	308 (61.1)
How old first wearing shoes (308)	≤ 15 years	6 (9.2)	127 (52.3)	133 (43.2)
	> 15 years	59 (89.8)	116 (47.7)	175 (56.8)
Where do you wear shoes (308)	At home	4 (6.1)	9 (3.7)	13 (4.2)
	On field	36 (55.4)	128 (52.7)	164 (53.3)
	Anywhere	25 (38.5)	106 (43.6)	131 (42.5)
Frequency of wearing shoes (308)	Daily	28 (43.1)	120 (49.4)	148 (48.0)
	Not daily	37 (56.9)	123 (50.6)	160 (52.0)
Type of shoe has (308)	Closed shoe plastic	23 (35.4)	88 (36.2)	111 (36.1)
	Closed shoe leather	16 (24.6)	37 (15.2)	53 (17.2)
	Closed canvas	16 (24.6)	21 (8.6)	37 (12.0)
	Sandals/Kongo	10(15.4)	97(40.0)	107 (34.7)
Wearing shoes at the time of interview (308)	No	15 (23.1)	52 (21.4)	67 (21.8)
	Yes	50 (76.9)	191(78.6)	241(78.2)
Type of shoe worn during interviewee (241)	Closed shoe plastic	15 (30.0)	72 (37.7)	87 (36.1)
	Closed shoe leather	13 (26.0)	21 (11.0)	34 (14.1)
	Closed canvas	14 (28.0)	19(9.9)	33(13.7)
	Sandals/Kongo	8 (16.0)	79 (41.4)	87 (36.1)
Availability of sufficient water	No	129 (76.3)	197 (58.8)	326 (64.7)
	Yes	40 (26.7)	138 (41.2)	178 (35.3)
Reasons for shortage of water (326)	Seasonal shortage	21 (16.3)	26 (13.2)	47 (14.4)
	Distance	108 (83.7)	171 (86.8)	279 (85.6)
Washing feet daily	No	148 (87.6)	215 (64.2)	363 (72.0)
	Yes	21 (12.4)	120 (35.8)	141 (28.0)
How to wash your feet	Water only	131 (77.5)	252 (75.2)	383 (76.0)
	Water and soap	38 (22.5)	83 (24.8)	121 (24.0)
Frequency by water only (383)	Daily	62 (47.3)	141 (56.0)	203 (53.0)
	Not daily	69 (52.7)	111 (44.0)	180 (47.0)
Frequency by water and soap (121)	Daily	10 (26.3)	37 (44.6)	47 (38.6)
	Not daily	28 (73.7)	46 (55.4)	74 (61.4)
Regular travel on barefoot	No	20 (11.8)	60 (17.9)	80 (15.9)
	Yes	149 (88.2)	275 (82.1)	424 (84.1)
Wear shoes during farming	No	144 (85.2)	245 (73.1)	389 (77.2)
	Yes	25 (14.8)	90 (26.9)	115 (22.8)
Barefoot farming time spent (389)	< 6 h	59 (41.0)	105 (42.9)	164 (42.2)
	≥ 6 h	85 (59.0)	140 (57.1)	225 (57.8)
Family history of leg swelling	No	102 (60.4)	324 (96.7)	426 (85.5)
	Yes	67 (29.6)	11 (3.4)	78 (14.5)

### Determinants of podoconiosis

In multivariable logistic regression analysis, variables like older age, male gender, low monthly income, having a pair of shoes, not washing feet daily, and family history were identified as determinants of podoconiosis. The odds of developing podoconiosis among those above 45 years of age were 3 times more likely compared to their counterparts [AOR: 3.05 (95% CI: 1.66, 5.60)]. The odds of developing podoconiosis in males were two-fold as compared to females [AOR: 2.03 (95%CI: 1.16, 3.55)]. The odds of developing podoconiosis among those who have a monthly income of ≤ 1000birr (≤ 7.16 USD) were more than 9 times more likely as compared to a monthly income of > 1000birr (> 7.16 USD) [AOR: 9.16 (95% CI: 4.97, 16.87)]. The odds of developing podoconiosis among participants who haven’t owned a pair of shoes were 3.3 times more likely compared to those who have owned a pair of shoes [AOR: 3.28 (95% CI: 1.77, 6.08)]. The odds of developing podoconiosis for those who hadn’t washed their foot daily were 2.93 times more likely compared to those who had washed their feet daily [AOR: 2.93 (95% CI: 1.22, 7.02)]. The odds of developing podoconiosis in those who have a family history of leg swelling were more than 21 times more likely as compared to those who haven’t a family history [AOR: 21.67 (95% CI: 9.21, 51.01)] (Table 4).

**Table 4:** Bi-variable and multivariable regression of determinants of podoconiosis at the age of 15 years and above in dera woreda, Northwest ethiopia, 2023 (*n* = 504)

Variables		Cases *N* (%)		Control *N* (%)	COR with 95% CI	AOR with 95% CI	*P*-value
Age	15–45 years	60 (35.5)	216 (64.5)		1	1	
	> 45 years	109 (64.5)	119 (35.5)		3.29 (2.24, 4.85)	3.05 (1.66, 5.60)	< 0.001
Sex	Male	117 (69.2)	190 (56.7)		1.72 (1.16, 2.54)	2.03(1.16, 3.55)	0.013
	Female	52 (30.8)	145 (43.3)		1	1	
Marital status	Single	22 (13.0)	30 (9.0)		1	1	
	Married	98 (58.0)	266 (79.4)		0.50 (0.28, 0.91)	0.60 (0.25, 1.41)	0.24
	Divorced	25 (14.8)	26 (7.8)		1.31 (0.60, 2.85)	0.74 (0.25, 2.19)	0.58
	Widowed	24 (14.2)	13 (3.8)		2.52 (1.05, 6.01)	2.25 (0.65, 7.79)	0.20
Education	No formal education	134 (79.3)	220 (65.7)		2.00 (1.29, 3.09)	1.05 (0.54, 2.05)	0.87
	Formal education	35 (20.7)	115 (34.3)		1	1	
Monthly income	≤ 1000 birrs (≤ 7.16 USD)	147 (86.9)	127 (37.9)		10.94 (6.64, 18.03)	9.16 (4.97, 16.87)	< 0.001
	> 1000 birrs (> 7.16 USD)	22 (13.1)	208 (62.1)		1	1	
Have owned a pair of shoes	No	104 (61.5)	92 (27.5)		4.22 (2.85, 6.25)	3.28 (1.77, 6.08)	< 0.001
	Yes	65 (38.5)	243 (72.5)		1	1	
Regular travel by barefoot	No	20 (11.8)	60 (17.9)		1	1	
	Yes	149 (88.2)	275 (82.1)		1.62 (0.94, 2.80)	1.14 (0.51, 2.55)	0.75
Availability of sufficient water to wash	No	129 (76.3)	197 (58.8)		2.26 (1.49, 3.42)	0.58 (0.27, 1.29)	0.18
	Yes	40 (26.7)	138 (41.2)		1	1	
Washing feet daily	No	148 (87.6)	215 (64.2)		3.93 (2.36, 6.54)	2.93 (1.22, 7.02)	0.016
	Yes	21 (12.4)	120 (35.8)		1	1	
Wear shoes during farming	No	144 (85.2)	245 (73.1)		2.11 (1.29, 3.45)	1.56 (0.75, 3.24)	0.23
	Yes	25 (14.8)	90 (26.9)		1	1	
Family history of leg swelling	No	102 (60.4)	324 (96.7)		1	1	
	Yes	67 (29.6)	11 (3.4)		19.35 (9.85, 38.01)	21.67 (9.21, 51.01)	< 0.001

## Discussion

This study was intended to address the determinants of podoconiosis. Variables like older age, male gender, low monthly income, not owning a pair of shoes, not washing feet daily, and a family history of leg swelling were statistically significant determinants. Older age participants were statistically significant for developing podoconiosis disease: study participants who were above 45 years were 3 times more likely to develop podoconiosis compared to their counterparts. This study is consistent with findings reported in Ethiopia, Uganda, Kenya, Rwanda, and Sub-Saharan Africa [2, 25, 27, 38, 39, 46, 47]. This is due to the fact that age increases the cumulative exposure to irritant soils and other risk factors, that enable a higher probability of acquiring disease. Moreover, the elderly suffer from physical, psychological, and mental weaknesses as the result of pathological changes that reduce and demotivate them to care for themselves [4]. In addition, elders may have less instruction/education on how to avoid the disease.

The sex of the participants was an exposing factor for podoconiosis: male participants had more than two-fold podoconiosis as compared to females. This result is similar to the study findings reported in the East and West Gojjam Zones [26]. This is due to the similarity of enabling exposure factors for podoconiosis in the study area. This finding is against other studies reported in Ethiopia, which revealed that females are more at risk of podoconiosis [6, 27, 32, 38, 40], whereas another study reported no sex difference [14]. The variation may not be sex differences but rather the result of prolonged exposure to risk factors, risk perception, and preventive behaviors like shoes wearing, and having access to high-quality shoes and socks, biological differences in disease prevention, gender inequality, and societal desires to maintain personal hygiene and self-care practices [27]. The geographical and environmental variation, such as low shoe-wearing practices, soil type (one-fourth of the Woreda has red clay soil), the majority rural agrarian community (86.3% of participants were farmers), and the male/female sex ratio of 1.6:1 may increase male exposure compared to previous studies. Moreover, males are more likely to engage in outdoor farming activities, and men are exposed to more risk factors over an extended period of time, which may raise their chance of suffering podoconiosis.

Participants’ income level was an important determinant of podoconiosis: study participants who had ≤ 1000-birr (≤ 7.16 USD) monthly income were more than 9 times more likely to develop podoconiosis compared to a monthly income of > 1000-birr (> 7.16 USD). This finding is consistent with previous studies conducted in the East and West Gojjam, Gamo, and Waghmra Zones [24, 26, 28]. This might result in a lack of adequate incentives or income to maintain a safe livelihood status (i.e., poor housing conditions, a lack of access to shoes and socks that require barefoot travel, poor self-care practices due to lack of soap and cleansing agents, and an inability to cover healthcare costs for healthcare services) at the family or individual levels. Moreover, podoconiosis affects the most productive age groups with chronic morbidity and disability, resulting in lost working days and social stigma that decrease economically productive life [14, 24, 28, 40, 48].

Similarly, study participants who did not have their own pairs of shoes were 3.28 times more likely to develop podoconiosis compared to their counterparts. This finding is supported by studies conducted in East and West Gojjam and Gamo Zones in Ethiopia and Kenya [24–26]. Shoe wear is not statistically significant in this study. Despite this, proper shoe-wearing prevents irritant soil exposure and other factors that protect from foot disfigurement and trauma [9, 14]. In addition, unprotected feet are exposed to red clay soils, which stimulates the lymphatic system, resulting in thickening and consequent lymphatic system obstruction, leading to podoconiosis [20].

Furthermore, participants who had not washed their feet daily were 2.93 times more likely to develop podoconiosis compared to those who washed their feet daily. This finding is similar to previous studies reported in Ethiopia and Sub-Saharan Africa [2, 20, 26–28, 32]. This is due to the prevention of dust, which causes superficial infections and damage by the irritant soil and surrounding environment, enabling them to maintain skin integrity and foot health. Finally, study participants with a family history of the disease were more than 21 times more likely to develop podoconiosis compared to those without a family history. This is consistent with findings reported in Wolayita and Gamo Zones, Bensa, Gulliso, and Machakel districts in Ethiopia [3, 5, 23, 37–39]. This resulted in evidence of heritability of genetic susceptibility to this specific disease in relation to sole nucleotide polymorphisms [4, 5, 8, 33].

This study highlights modifiable factors like monthly income level, owned shoes, and regular foot washing are the enabling factors to prevent podoconiosis. However, older age and a family history of leg swelling are predisposing factors. To eliminate this neglected tropical disease, cost-effective interventions such as subsidized shoes, implementing community-led surveillance using family history as a screening tool, and strengthening personal hygiene behavioral changes by health extension workers are required.

### Strengths and limitations of the study

This study was conducted in a community-based with a relatively large sample size, which supports the findings’ generalizability. Neighbor controls are selected at random, resulting in a lack of representativeness of cases in relation to reality (i.e. due to differences in podoconiosis prevalence of ages and sex). Unmatched study designs allow for variation between cases and controls, but they may not be able to control potential confounding factors that could alter the findings. There is also a weak ability to ascertain the antecedent of the relationship in a case-control study. In addition, there may be also information biases like recall and interviewer biases from previous exposures and shared household exposures (e.g., barefoot practices, water access) may have an effect on the finding as the study’s limitations.

## Conclusions

In this study, older age, male gender, low monthly income, not owning a pair of shoes, not washing their feet daily, and a family history of leg swelling were identified as determinants of podoconiosis. Encouraging shoe ownership, foot-washing practices, and rising income levels are very important measures for controlling podoconiosis. Health extension workers, healthcare professionals, and other responsible stakeholders should improve and strengthen health education and health promotion activities. Adopting and implementing community-led surveillance using family history as a screening tool is pivotal to finding undiagnosed cases. Furthermore, the government also focuses on cost-effective interventions like shoe subsidies and improving water and sanitation facilities to eliminate this neglected tropical disease nationwide. Further prospective and matched case-control studies that account for environmental factors will be encouraged.

## Supplementary Information

Supplementary Material 1.

Supplementary Material 2.

## Data Availability

The datasets used and/or analyzed during the current study are available from the corresponding author upon reasonable request.

## Abbreviations

AOR: Adjusted Odds Ratio
CI: Confidence Interval
COR: Crude Odds Ratio
HEW: Health Extension Workers
NTDs: Neglected Tropical Diseases
WHO: World Health Organization
