# Supplementary figures and images for: Determinants of podoconiosis at the age of 15 years and above at Dera Woreda, South Gondar zone, Northwest Ethiopia
Source: BMC Public Health. 2025 Oct 6;25:3359. doi: 10.1186/s12889-025-24723-8 (PMC12502329; doi:10.1186/s12889-025-24723-8)

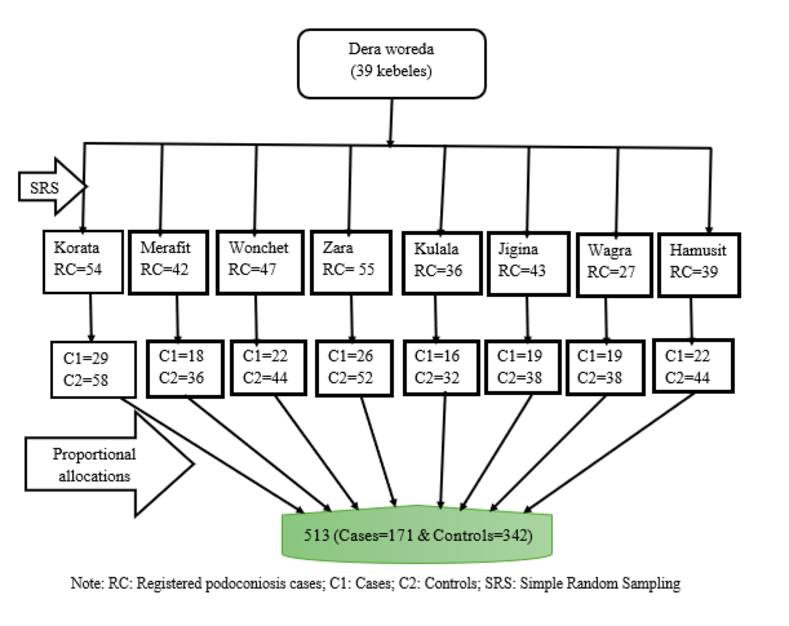

Supplement: Supplementary file 1 — Supplementary Material 1. [file 12889_2025_24723_MOESM1_ESM.tif]
