# Supplementary material for: Determinants of podoconiosis at the age of 15 years and above at Dera Woreda, South Gondar zone, Northwest Ethiopia
Source: BMC Public Health. 2025 Oct 6;25:3359. doi: 10.1186/s12889-025-24723-8 (PMC12502329; doi:10.1186/s12889-025-24723-8)
Supplement: Supplementary file 2 — Supplementary Material 2. [file 12889_2025_24723_MOESM2_ESM.docx]

**Data collection tool English version**

Participant code --------------

Data collector Name ______________________ Signature ________ Date _____________

| **No** | **Variables** | **Characteristics** | **Skip** |
| --- | --- | --- | --- |
| **Part I** | **Socio-demographic characteristics** |  |  |
| 101 | Participant age | ----------- years |  |
| 102 | Sex of participant | 1. Male 2. Female |  |
| 103 | What is your current marital status? | 1. Single 2. Married 3. Divorced 4. Widowed |  |
| 104 | What is your religion? | 1. Orthodox 2. Muslim 3. Protestant 4. Others ______ |  |
| 105 | Educational status? | 1. No formal education 2. Formal education |  |
| 106 | Occupation status? | 1. Farmer 2. Merchant 3. Daily laborer 4. Others ______ |  |
| 107 | How much is your monthly income? | -------------- Birrs |  |
| **Part II** | **Behavioral and practice-related factors** |  |  |
| 201 | Do you think podoconiosis can be preventable? | 1. Yes 2. No |  |
| 202 | If the answer “yes” how can podoconiosis be preventable? | 1. Wearing shoes 2. Washing feet regularly 3. Avoiding marriage with affected people 4. Avoiding contact with affected people |  |
| 203 | If the answer “no” why podoconiosis not prevented? | 1. Genetics 2. No drugs 3. Not seen cured people 4. Others -------- |  |
| 204 | Have you ever owned a pair of shoes? | 1. Yes 2. No | If 2 skip to 211 |
| 205 | How old were you when you first owned a pair of shoes? | ----------Years |  |
| 206 | Where do you wear shoes? | 1. At home 2. On the field 3. Anywhere |  |
| 207 | Frequency of wearing shoes | 1. Daily 2. Not daily |  |
| 208 | What type of shoes do you have? | 1. Closed shoes plastic 2. Closed shoes leather 3. Closed Canvas 4. Sandals/Kongo |  |
| 209 | Wearing shoes at the time of interview | 1. Yes 2. No |  |
| 210 | What kind of shoes does the interviewee wear at the time of interview? | 1. Closed shoes plastic 2. Closed shoes leather 3. Closed Canvas 4. Sandals/Kongo |  |
| 211 | Can you get enough water to wash your feet? | 1. Yes 2. No | If 1 skip to 213 |
| 212 | What is the problem? | 1. Seasonal shortage 2. Distance 3.Other(specify): ____ |  |
| 213 | Do you wash your feet daily? | 1. Yes 2. No | If 2 skip to 301 |
| 214 | How do you wash your feet? | 1. By water only 2. By water and soap | If 2 skip to 216 |
| 215 | Frequency of feet washing by water only? | 1. Daily  2. Not daily |  |
| 216 | Frequency of feet washing by water and soap? | 1. Daily  2. Not daily |  |
| Part III | **Exposure and family related factors** |  |  |
| 301 | Do you regularly travel barefoot for different social purpose? | 1. Yes  2. No |  |
| 302 | During farming do you wear shoes? | 1.Yes  2. No |  |
| 303 | How much time do you spend farming barefoot? | ------- Hours |  |
| 304 | Is there any family member have leg swelling? | 1. Yes 2. No |  |
| 305 | Whose family member is/are affected? | 1. Father 2. Mother 3. Grandparents 4. Others ------------ |  |

**Thank you very much for your time and participation!**
